# From Clinic to Home: Empowering Parents with Naturalistic Developmental Behavioral Interventions for Autism Spectrum Disorders

**DOI:** 10.2174/0117450179451314260330102440

**Published:** 2026-03-31

**Authors:** Sergio Machado, Flávia Paes, Hellen Condal, João Lucas Lima

**Affiliations:** 1 Center for Neuroscience, Neurodiversity Institute, Nova Iguaçu-RJ, Brazil; 2 LABPR – Panic and Respiration Laboratory (LABPR) - Institute of Psychiatry (IPUB), Federal University of Rio de Janeiro (UFRJ), Rio de Janeiro, Brazil; 3 Virtual Reality in Mental Health Laboratory (RVSM) - Institute of Psychiatry (IPUB), Federal University of Rio de Janeiro (UFRJ), Rio de Janeiro, Brazil; 4 Atipicamente ABA, Rio de Janeiro, Brazil

## 1 INTRODUCTION

The potential of programs that empower parents to intervene in the development of children with autism (ASD) is unquestionable. When parents become protagonists in the intervention, therapeutic support becomes a natural part of daily life. This approach promotes skill generalization and increases intervention intensity in a sustainable and cost-effective manner [1,2]. We are facing a paradigm shift; instead of the traditional clinic-centered model, learning opportunities are incorporated into the family's daily routines and interactions [2]. However, for clinicians and families navigating a field replete with methodologies from the comprehensive Early Intervention Denver Model (ESDM) and Pivotal Response Treatment (PRT), which focus on crucial skills, to the structured coaching of ImPACT (Improving Parents as Communication Teachers) and the engagement-based approach of JASPER (Joint Attention, Symbolic Play, Engagement, and Regulation), the central question remains: how to define the most appropriate starting point?

Ouyang and colleagues’ sophisticated network meta-analysis [3] advances the discussion by making a crucial contribution: it goes beyond the generic question of effectiveness and moves toward a detailed, data-driven understanding of comparative effectiveness. Their results do not simply confirm that Naturalistic and Developmental Behavioral Interventions (NDBIs) work; they point to a smarter, more personalized, and sequential approach to delivering them, tailored to each family's individual needs and priorities. Building on this comparative approach, the evidence suggests that clinical goals should guide intervention selection. If the primary clinical goal is to accelerate a child’s language and motor development directly and efficiently, ESDM emerges as a particularly powerful tool. Ouyang *et al.* [3] found ESDM to be specifically effective in these critical domains compared to TAU (SMD = 0.41 for language; SMD = 0.44 for motor skills), a result strongly corroborated by earlier seminal studies [4,5]. This suggests a logical clinical pathway: after establishing basic parental fidelity and confidence through an initial program like ImPACT, transitioning to a more comprehensive, child-directed model like ESDM could maximize developmental gains across broader areas, consistent with the implementation science literature on staged intervention approaches [3].

## 2 THE STEPPED-CARE MODEL

For children who continue to struggle with core social communication challenges despite foundational support, PRT may represent the highest tier of intervention. The network meta-analysis ranked it highest for improving both social (74.1%) and language (88.3%) skills [3], but with a critical caveat: its success is inextricably linked to achieving high parent fidelity, which, in turn, requires significant training intensity and support [6]. This makes PRT less suitable as a universal starting point but potentially transformative for families who have already mastered foundational coaching strategies and are prepared for a more intensive, pivotal behavior-focused approach, as demonstrated in advanced parent training studies [7]. The clear variation in intervention strengths and prerequisites naturally leads to the question of how to systematically organize their delivery. This is where the concept of a structured care model becomes essential. The evidence on the comparative effectiveness of interventions strongly supports the adoption of a stepped-care model [2]. This model ushers in a new way of thinking, abandoning the standardized approach in favor of a dynamic, adaptable, and resource-optimizing system, which aligns with contemporary healthcare delivery models. Guided by a prioritization logic, interventions are sequenced based on each family's specific strengths and evolving needs and capabilities, resulting in a truly personalized pathway [8].

Within this framework, ImPACT is not seen as a competitor to ESDM or PRT, but rather as their logical and indispensable precursor [3]. It serves as an effective first step, providing parents with the foundational skills and confidence needed for the process. Its structured, manualized nature is ideal for building basic competency, making it the most appropriate starting point for most families, particularly for those new to therapeutic techniques [9]. Success in this phase is measured by significant gains in parents' implementation fidelity, which serves as the foundation for subsequent steps [4]. Once this foundation is solidified, ESDM can be introduced as a second step to promote development more broadly. Leveraging parents' newfound skills in naturalistic engagement, the comprehensive ESDM curriculum targets a broader range of outcomes, such as language and motor skills, within daily routines. This stage builds parents' confidence to address developmental goals more holistically, capitalizing on the established parent competence [10].

For children who continue to exhibit core social communication challenges even after this initial support, PRT is reserved as a third, high-intensity stage. This stage specifically targets pivotal areas such as motivation and self-initiation. The model recognizes that the success of PRT depends on an advanced level of parenting skill, precisely what the previous stages aim to develop. Introducing PRT only after proficiency in fundamental strategies prevents early abandonment and maximizes the potential for transformative outcomes, consistent with findings from studies on treatment sequencing [11].

This sequential model respects the family's evolving capacity. The model is patient- and family-centered, introducing complexity gradually so as not to overwhelm parents from the outset. This gradual progression in intensity is crucial for ensuring long-term adherence, reducing family stress, and increasing satisfaction with the intervention [2]. By matching intervention intensity to family need and readiness, the stepped-care model represents a transition from a static allocation of intervention to a dynamic, efficient, and more equitable system, promoting better resource utilization [2].

The study by Ouyang *et al.* [3] also highlights a persistent challenge in the field of parent-mediated interventions: the pressing need for standardized, validated fidelity measurement tools [12]. Fidelity is a crucial mediator of outcomes, but its assessment remains inconsistent across studies and clinical settings, as noted in implementation science research [13]. The effectiveness of the stepped-care model depends entirely on the ability to accurately assess parents' fidelity at each stage to determine the transition point. In addition to the limitations already mentioned, it is important to consider the inherent restrictions of network meta-analysis design. The validity of indirect comparisons and effectiveness rankings presupposes the absence of inconsistencies and the transitive comparability of the included studies. It is fair to point out that our review encounters a common limitation in this field: the diversity of studies. Children with different profiles received interventions of varying intensity and duration, measured with different instruments. Furthermore, with most of the data originating from the USA, it is worth asking: would the same benefits be maintained in countries with different family structures and public support? Culture is a fundamental ingredient in family dynamics, and it inevitably influences how parents receive and apply intervention strategies. Although we have employed random-effects models and sensitivity analyses to mitigate some of these issues, these sources of heterogeneity should be considered when interpreting our findings.

## 3 IMPLEMENTATION FIDELITY AS A CORE CHALLENGE

The significant variation in implementation fidelity observed across interventions and studies serves as an important warning: the key to the success of these programs lies not solely in the manual's content, but rather in the quality, consistency, and skill with which parents apply the strategies in their daily routines. This inconsistency directly threatens the internal and external validity of scientific findings. In the absence of reliable methods for measuring implementation, it becomes extremely difficult to isolate the true effect of the intervention, distinguish between an ineffective model and an effective one applied inappropriately, or identify which components produce changes, a difficulty widely recognized in the intervention research literature [14].

The problem is compounded by the lack of standardization in the field, which constitutes a significant obstacle to comparative effectiveness studies. When studies use different and often unvalidated fidelity scales, accurate comparison of results across studies or synthesis of evidence in meta-analyses becomes virtually impossible. This current scenario makes it difficult to understand which interventions work, for which child profiles, and in which contexts, consequently slowing the advancement of evidence-based practice [15].

## 4 CLINICAL AND RESEARCH IMPLICATIONS

The implications extend decisively to clinical practice. For therapists, the lack of a unified instrument complicates the process of supervising and guiding parents. This limits their ability to provide accurate, data-driven feedback, personalize support, and make informed decisions about transitioning to more complex intervention models. For families, this gap can translate into missed opportunities for developing specific child skills and, potentially, suboptimal outcomes, highlighting the clinical consequences of current measurement limitations [16]. Given this context, it is imperative that future research prioritize the development and validation of unified fidelity instruments that are simultaneously psychometrically robust and practical for real-world use.

These tools must be able to capture the core components of NDBIs, such as environmental arrangement, responsive interaction, and naturalistic teaching episodes, while also being sensitive enough to detect nuances in the quality of implementation. Investing in this area of measurement is not a secondary concern, but a fundamental prerequisite for advancing the field, as emphasized in measurement science literature [17]. This will enable a more precise understanding of the mechanisms of change, facilitate the replication of successful results, and ultimately ensure that the potential of parent-led interventions is fully realized by each family.

The strategic integration of digital health tools presents a promising pathway to address these implementation challenges and operationalize the stepped-care model. Telehealth platforms can dramatically increase access to initial parent coaching and ongoing supervision, particularly for families in remote or underserved areas [3]. Furthermore, mobile applications can support parents in daily practice by providing just-in-time reminders of strategies, facilitating progress tracking, and offering structured video examples, effectively functioning as a portable fidelity support system. Perhaps most innovatively, emerging technologies such as Virtual Reality (VR) have the potential to create controlled yet ecologically valid training environments where parents can safely practice and refine their intervention skills before applying them in complex real-world interactions with their child [18]. By leveraging these technologies, we can not only mitigate the current barriers of cost and access but also enhance the quality, scalability, and personalization of parent-mediated interventions, making the journey from clinic to home more efficient and effective.

## CONCLUSION

In summary, contemporary systematic reviews, such as the one conducted by Ouyang *et al.* [3], go beyond simply prioritizing interventions; they outline a pragmatic clinical roadmap. The integration of strategic selection, a phased care architecture, and rigorous fidelity assessment allows us to overcome the fruitless search for a single “ideal” intervention. By adopting a strategic, sequential, and deeply personalized parent coaching approach, we can optimize the use of limited resources and respect each family's learning pace. The result is the provision of more individualized, sustainable, and effective care for children with autism, truly extending support far beyond the confines of the therapy room, representing the future direction of autism intervention services [19]. Ultimately, this integrated vision, where evidence-based protocols are dynamically tailored to individual family needs, heralds a new paradigm in autism care: one that is not only more personalized and ecological but also profoundly more empowering and sustainable.

## LIST OF ABBREVIATIONS

PRT: Pivotal Response Treatment
JASPER: Joint Attention, Symbolic Play, Engagement, and Regulation
NDBIs: Naturalistic and Developmental Behavioral Interventions
VR: Virtual Reality
